# Stabilization of directly acidified protein drinks by single and mixed hydrocolloids—combining particle size, rheology, tribology, and sensory data

**DOI:** 10.1002/fsn3.1933

**Published:** 2020-10-15

**Authors:** Jing Liu, Heidi Liva Pedersen, Lisbeth Knarreborg, Richard Ipsen, Wender L. P. Bredie

**Affiliations:** ^1^ Department of Food Science Faculty of Science University of Copenhagen Rolighedsvej 26 1958 Frederiksberg C Denmark; ^2^ CP Kelco Ved Banen 16 4623 Lille Skensved Denmark

**Keywords:** acidified milk drinks, hydrocolloids, rheology, sensory analysis, tribology

## Abstract

**Background:**

High methoxyl pectin and carboxymethylcellulose (CMC) can be used as a stabilizer for directly acidified protein drinks (DAPDs). Use of pectin or CMC together with other polysaccharides and their impacts on product's rheological properties and tribological behavior are still largely unknown. This project investigated the impact of pectin and CMC, alone or in combination with guar gum, locust bean gum (LBG), and gellan gum when preparing DAPDs. The particle size distributions, rheological properties, tribological properties, and sensory properties were determined.

**Results:**

Pectin and CMC were dominating in the mixed system with other stabilizers. Increasing the concentration of hydrocolloids resulted in higher viscosity and better lubrication (lower friction coefficient). The sensory *viscosity*, *smoothness*, *coating*, and *stickiness* intensified as the concentration of hydrocolloids increased. The type and amount of hydrocolloids had a strong effect on the sensorial texture perception, but the flavor perception was only slightly affected.

**Conclusion:**

Use of combined stabilizers may contribute to providing an effective viscosity enhancement without affecting the flavor in acidified milk beverages.

## Graphical abstract

The rheological, tribological, and sensory properties of directly acidified protein drinks (DAPDs) were studied. Increasing the concentration of hydrocolloids resulted in higher viscosity and better lubrication (lower friction coefficient). The type and amount of hydrocolloids had a strong effect on the sensorial texture perception, but the flavor perception was only slightly affected.

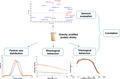

## 1 INTRODUCTION

Acidified protein drinks (APDs) are popular food products found worldwide. Many types of APDs are available, including drinking yogurt, beverages containing both milk and fruit juice, and soft drinks containing milk solids as a minor ingredient (Janhøj et al., 2008; Jensen et al., 2010). Such drinks are usually manufactured by direct acidification or fermentation of milk with lactic acid bacteria, followed by homogenization and typically a final heat treatment to extend shelf life (Amice‐Quemeneur et al., 1995; Laurent & Boulenguer, 2003). A common trait of APDs is their low pH at around pH 4.0, which results in sedimentation problems due to aggregation of milk proteins. To avoid the flocculation of milk proteins and subsequent macroscopic whey separation, addition of a protein‐protecting stabilizer to APDs is usually required (Tromp et al., 2004).

Choice of the proper hydrocolloid type and concentration is one of the most essential decisions in control of the physical and sensory quality of APDs. Furthermore, it is important that the hydrocolloids do not mask the natural flavor (Gallardo‐Escamilla et al., 2007). In the present study, six hydrocolloids were used, including pectin and two types of carboxylmethylcellulose (CMC) that are commonly used in APDs, as well as gellan gum, guar gum, and locust bean gum (LBG) that are gradually being introduced in dairy products. In all samples, either pectin or CMC was added in concentrations which ensured protein protection (i.e., avoiding phase separation) during the final heat treatment. Gellan gum, guar gum, and locust bean gum were added to enhance texture and/or stability of the final drink. Pectin is widely used in many dairy products as a stabilizing agent. Pectin molecules interact with casein through calcium ions and prevent their aggregation and sedimentation, and hence prevent serum separation by ionic and steric stabilization of protein in acidic milk beverages (Koksoy & Kilic, 2004). CMC is used in APDs in some markets because of tradition and economy as it, although being less efficient than pectin, it ensures stabilization by a similar mechanism. During acidification, CMC has been demonstrated to adsorb onto the casein micelles by electrosorption and the adsorbed CMC layer on the surface of casein can prevent aggregation and sedimentation of casein micelles by electrostatic and steric stabilization. In addition, nonadsorbed CMC can slow down sedimentation of casein particles by increasing viscosity of the continuous phase (Du et al., 2007; Wu et al., 2014). Both guar gum and LBG act as viscosity enhancers in APDs; they have no casein‐protecting properties and a second hydrocolloid such as pectin is therefore needed when the APD is heat treated to ensure long shelf life. Gellan gum does not protect proteins either, but is used in order to create a network/fluid gel which supports the stability by suspending proteins protected by other hydrocolloids.

The behavior of hydrocolloids in food can be examined using both instrumental and sensory measures. A traditional method to assess the mouthfeel of a fluid is measuring viscosity as a function of shear rate, usually between 10 s^−1^ and 1,000 s^−1^ (Shama & Sherman, 1973). Numerous studies have been performed using rheological measurements (Javidi et al., 2016; Sharma et al., 2017). However, it is evident that rheology alone cannot predict complex sensory parameters such as mouth coating or creaminess. For this reason, food tribology has emerged as a new experimental technique alongside food rheology for investigations of the relationship between food structure, texture, and mouthfeel (Chen & Stokes, 2012; Prakash et al., 2013; Sarkar & Krop, 2019). Tribology (also known as thin film rheology) provides important information on material properties in the form of thin film. During oral processing, food acts as a lubricant; hence, the frictional forces during in‐mouth processing depend on the food properties. Only a few recent studies have been conducted to evaluate the tribological properties and their associations with corresponding sensory notes of dairy products (Laguna et al., 2017; Laiho et al., 2017; Nguyen et al., 2017), hydrogels (Krop et al., 2019), or emulsions (Upadhyay & Chen, 2019). These studies indicate that rheology and tribology may reflect different aspects of oral behavior and correlate with specific sensorial attributes or different time points of the eating process (Chen & Stokes, 2012).

Research in combining two or more polysaccharides to stabilize APDs is still limited. Therefore, the objective of this study was to investigate the rheological, tribological, and sensory properties of pectin, CMC and a combination with several other hydrocolloids in acidified milk systems.

## 2 MATERIALS AND METHODS

### 2.1 Materials

The hydrocolloids used in this study were as follows: a high methoxyl pectin (DE of 69%, experimental pectin, CP Kelco, Lille Skensved, Denmark), two types of carboxymethyl cellulose (CMC A: CEKOL^®^ 30, CP Kelco, Äänekoski, Finland, substitution degree of 0.84; CMC B: CEKOL^®^ 4000, CP Kelco, Äänekoski, Finland, substitution degree of 0.82), guar gum (E412, Acatris Netherlands B.V., Netherland), locust bean gum (GENU^®^ Gum Refined LBG, CP Kelco, Lille Skensved, Denmark) and gellan gum (KELCOGEL^®^ HM, CP Kelco, Lille Skensved, Denmark). Skim milk powder (34% protein) was obtained from Uhrenholt, Denmark. All materials were used without further purification. Demineralized water was used in preparation for the samples. All samples for sensory evaluation were prepared under food‐grade conditions.

### 2.2 Sample preparation

The present work focused on a type of APDs with direct acidification, namely directly acidified protein drinks (DAPDs). Twelve DAPDs were produced by adding pectin and two types of CMC, either alone or in combination with guar gum, LBG, or gellan gum. All hydrocolloids were added in two concentrations. The reason for the selection of hydrocolloids and levels was to achieve commercial relevance as well as a wide span of sensory properties. The used hydrocolloids along with the concentrations have been displayed in Table 1. For each drink, the stabilizer was dissolved the day before in deionized water to obtain a 1% stabilizer solution. Pectin and pectin blends were dissolved at 60°C in deionized water by means of a Silverson L4R (Silverson Machines Inc. East Longmeadow, MA 01028, USA) and then cooled to 5°C. All CMC samples were dissolved with an IKA propeller stirrer at ambient temperature and then cooled to 5°C. Stabilizer solutions were stored cold (5°C) overnight.

**TABLE 1:** Types and concentrations of hydrocolloids added to DAPDs

No.	Code	Hydrocolloid	% w/w
1	Pectin_low	Pectin	0.20
2	Pectin_high	Pectin	0.40
3	Pectin/Guar_low	Pectin/Guar gum^*^	0.21/0.14
4	Pectin/Guar_high	Pectin/Guar gum^*^	0.33/0.22
5	Pectin/LBG_low	Pectin/LBG^*^	0.21/0.14
6	Pectin/LBG_high	Pectin/LBG^*^	0.33/0.22
7	CMC A_low	CMC A	0.65
8	CMC A_high	CMC A	0.85
9	CMC B_low	CMC B	0.45
10	CMC B_high	CMC B	0.65
11	CMC/Gellan_low	CMC/Gellan gum^*^	0.80
12	CMC/Gellan_high	CMC/Gellan gum^*^	1.00

^*^ The ratio of pectin/guar gum, pectin/LBG was 3 to 2; the ratio of CMC/gellan gum was confidential.

To make DAPDs, 9.0% high‐soluble skim milk powder and 21.2% sucrose were dry‐blended and mixed with 69.8% deionized water. After agitating for 10 min, the sweetened milk base was further hydrated for 30 min at ambient temperature without stirring. The sweetened milk base was blended with various concentrations of a 1% stabilizer solution to obtain stabilizer concentrations as in Table 1. The pH of all samples was adjusted to pH 4.0 with citric acid solution (30% w/w), and the total weight was adjusted with deionized water. The drinks were homogenized and heat treated on a MicroThermics UHT/HTST DIPW installation (MicroThermics, Inc., 3216‐B Wellington Ct., Raleigh, NC 27615, USA). Samples were homogenized at 180–200 bar (ambient temperature, no preheat), heat treated at 121°C for 4 s, and cooled to 20°C. Finally, the drinks were filled aseptically into 200 ml PET bottles and stored at 23°C. All instrumental measurements and sensory analysis were performed within one week after sample production, and samples were turned upside down ten times before all measurements.

### 2.3 Particle size measurements

The particle size distribution of the DAPDs was determined by laser diffraction using a Mastersizer 2000 (Malvern Instruments Ltd, Worcestershire, UK) and deionized water as the dispersion media in the water bath of the instrument. The sample was added to the water bath using a disposable pipette until an obscuration slightly exceeding 10 units was reported by the instrument software. Measurements during 12 s (12,000 snaps) were performed twice for each of above dispersions. The measurements were interpreted as particle size distributions by assuming the following parameters: the refractive index of dispersant (water) = 1.33, refractive index of dispersed phase (protein/stabilizer “particles”) = 1.3484, the absorbance index = 0.00021 (Calhoun et al., 2010). An averaged distribution is reported for each sample.

### 2.4 Rheological measurement

Viscosity of the samples was measured at 23°C with an Anton Paar MCR 101 rheometer (Anton Paar GmbH, 8054 Graz, Austria) equipped with a cylindrical double gap geometry (DG26). 8 ml of samples was placed in the rheometer cup with a disposable pipette while avoiding air bubbles. The samples were then measured with a program with continuous rotation starting at shear rate 10 s^−1^ and recording one measurement for each five seconds until having collected six measurements. Same program then stepped up and later down until having covered (in the order of listing) the shear rates 10, 30, 100, 300, 1,000, 300, 100, 30, 10 s^−1^ with six measurements each. Viscosity is reported for each sample as a function of shear rate (s^−1^) and averaged across six replicates across two measurements.

### 2.5 Tribological measurement

Tribological behaviors of the samples were measured at 23°C with an Anton Paar MCR 301 rheometer (Anton Paar GmbH, 8054 Graz, Austria) equipped with a tribology cell (BC geometry), a glass ball and PDMS pins (SP‐BC6‐6/PDMS). 1.5 ml of samples was placed in the cell avoiding air bubbles. Since in‐mouth force has been reported to be between 0.01 and 10 N (Miller &Watkin, 1996), we used a constant normal force of 3 N to represent the moderate normal force applied on the sample during oral processing. The sliding speed was increased from 0.1–1000 mm s^−1^ (300 measuring points, 1s per measuring point). The tribology cell and glass ball were cleaned with deionized water and then rinsed with 100% IPA and dried with paper towel. Air spray was used to dry completely before adding next sample. The friction coefficient, calculated as the ratio of the measured friction force against the normal load, was reported for each sample as a function of sliding speed (mm/s). The measurement was carried out in two replicates, and average values are reported.

### 2.6 Sensory analysis

To obtain a sensory profile of the samples, a sensory descriptive analysis was performed in the Future Consumer Lab of University of Copenhagen. An external trained panel consisting of nine female assessors (mean age = 30.4 ± 5.8) was recruited. All assessors had been screened for sensory acuity and availability prior to inclusion in the sensory panel and were experienced in sensory evaluation of food (Liu et al., 2015, 2018). All assessors were compensated for their participation. The present study was performed according to the principles established by the Declaration of Helsinki.

Four training sessions were held in order to develop the vocabulary and get the assessors familiar with the samples. The assessors were firstly provided with a set of samples and asked to generate attributes freely. The panel leader wrote down all attributes on a white board and a list of pooled attributes was thus obtained. Afterward, reference standards for the pooled attributes were presented to the panel, discussed, and modified. The list of final sensory attributes along with their definition and references has been displayed in Table 2. Among the attributes, five were relating to texture in‐mouth, two were relating to basic taste, one was relating to mouthfeel, three were relating to flavor, one was relating to aftertaste, and one was relating to overall flavor intensity. Evaluation was conducted in individual sensory booths at room temperature (20 ± 1°C). Twelve samples were assessed in four replicates, which were held in different days. All samples were presented in a different order specific to each assessor according to a Williams Latin‐square. Each time, 50 g of sample in plastic cup was presented to the panel. The cups were coded with 3‐digit numbers and covered with plastic caps. The assessors were asked to scale each attribute for all the samples in an unstructured, 15‐cm linear scale in the FIZZ system (version 2.51A, Biosystems, Courtenon, France). Water and unsalted crackers were provided for cleaning the palate between tasting.

**TABLE 2:** Sensory attributes with corresponding definitions, scale anchors, and reference materials

Modality	Attribute	Definition	Scale	Reference material
Texture	Viscosity	The pressure taken to move the sample between the tongue and the palate	Thin → thick	Low fat milk 0.1% to light whipped cream
	Smoothness	Velvety feeling of the sample in the mouth	None → a lot	Milkshake
	Powdery	Powdery sensation	None → a lot	3 g potato starch in 300 ml milk (1.5% fat)
	Stickiness	Degree to which the sample sticks to the teeth and palate	None → a lot	Milkshake
	Coating	The amount of sample remaining in mouth after swallowing	None → a lot	50% chocolate + 50% of cream (38% fat)
Mouthfeel	Astringent	Trigeminal sensation of drying, drawing, puckering of the mouth surfaces	None → a lot	2 tea bags in 500 ml boiled water for 10 min
Basic taste	Sweet	Taste on the tongue stimulated by sugars	A little → a lot	24.0 g/L sucrose in water
	Sour	Taste on the tongue stimulated by acids	A little → a lot	Buttermilk (0.5% fat)
Flavor	Citrus	Flavor sensation associated with lemon, lime	None → a lot	Mixed lemon and lime
	Fruity	Flavor sensation associated with mixed fruits	None → a lot	Yogurt with mixed fruits
	Buttermilk	Flavor sensation associated with butter milk	None → a lot	Buttermilk (0.5% fat)
Aftertaste	Metallic	Aftertaste sensation associated with the metal	None → a lot	0.016 g/L iron sulfate in water
	Overall intensity	Intensity in general when assessing the sample	None → a lot	

### 2.7 Statistical analysis

The instrumental data were assessed by a one‐way analysis of variance (ANOVA) with product as independent variables and instrumental measurements as dependent variables. When significant product effects were found, the ANOVA was followed by post hoc comparison by Tukey's Honest Significant Difference (HSD) test (*p* < .05).

The sensory data were assessed by ANOVAs using a mixed model with product and replicate as fixed effects and judge as random, followed by pairwise comparisons by Tukey HSD test. To enable a visual exploration of the sensory results, principal component analysis (PCA) was conducted on the significant sensory attributes using data averaged across both replicates and judges. The data were mean‐centered and standardized (1/Sdev) prior to running the PCA model. Pearson's correlations between the sensory and instrumental measurements for particle size, rheology and tribology were calculated from the average sample data.

The ANOVAs and correlation models were run using the software IBM SPSS Statistics 25 (IBM Corporation, Armonk, NY, USA). The PCA was performed using the Unscrambler version 10.5.1 (CAMO ASA, Oslo, Norway).

## 3 RESULTS AND DISCUSSION

### 3.1 Particle size

**FIGURE 1:**
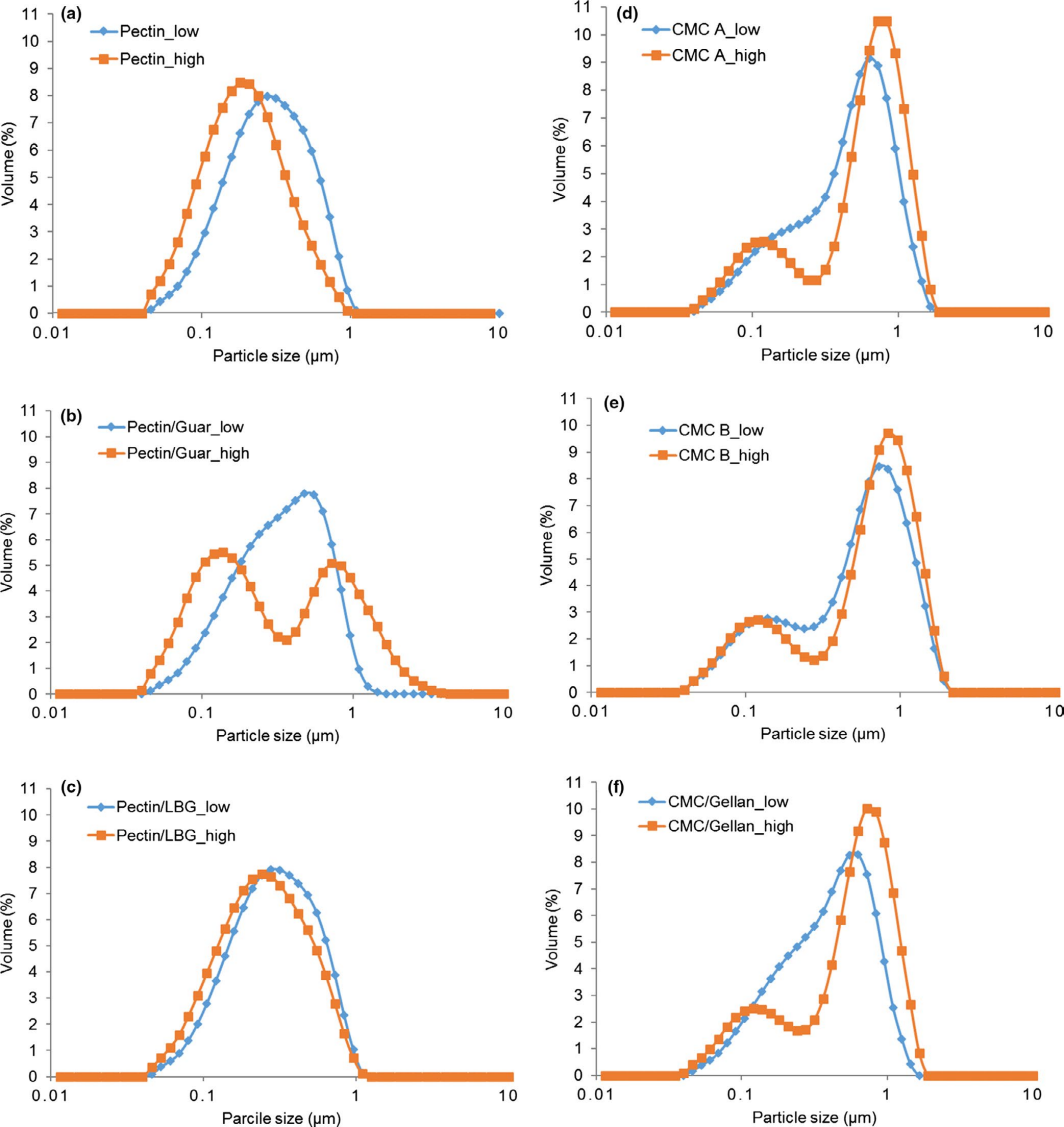
Particle size distributions of DAPDs added with pectin, pectin/guar gum, pectin/LBG, CMC A, CMC B, and CMC/gellan gum in low concentration (blue line) and high concentration (orange line)

The particle size distributions of DAPDs are shown in Figure 1. As can be seen, the particle sizes of pectin added samples were reduced as the concentration of stabilizers increasing. Previous research has also reported that increasing pectin concentration result in a decreased particle size in acidified milk beverages (Lucey et al., 1999; Sedlmeyer et al., 2004). Contrarily, CMC samples exhibited a shift toward larger average particle size when the levels of stabilizers increased. This is in agreement with the work of Ntazinda et al. (2014) who reported that increasing CMC concentration above a certain level (to 6 g/L) caused a broadening of the particle size peak toward larger sizes. Yuliarti et al. (2019) also found increased particle size in systems with CMC added with a small amount of pectin. The increase in particle size with increased CMC could possibly be a limited depletion flocculation effect, but it should be noted that the changes of particle size were minor in all systems.

Generally, when the concentration of stabilizers was at low level, all samples exhibited monomodal particle distributions. As the concentration of stabilizers was increased, CMC samples as well as samples with pectin/guar gum exhibited double peaks, presumably a peak of stabilized casein aggregates and another one consisting of nonstabilized micelles. Previous research indicates that adsorption of a CMC layer on the surface of these micelles prevents flocculation by steric repulsion forces (Du et al., 2007). The double peak might be also attributed to the limited depletion flocculation effect. Tuinier and de Kruif (2002) found that mixing guar gum with casein micelles resulted in phase separation due to depletion interaction causing an effective attraction between the casein micelles by nonadsorbing guar gum; however, the pH was 7 in their case. It is also worth considering how the method of addition affects the stabilization; the stabilizers were added prior to acidification and one could assume some self‐aggregation occurred, especially at high level, hence a population of unstabilized casein micelles emerged. Komorowska et al. (2017) demonstrated that the polymer chains may be aggregated by hydrogen bonds in aqueous solutions of sodium CMC. Such inherent aggregation would influence functionality and affect the ability to interact with casein micelles.

### 3.2 Rheological behavior

**FIGURE 2:**
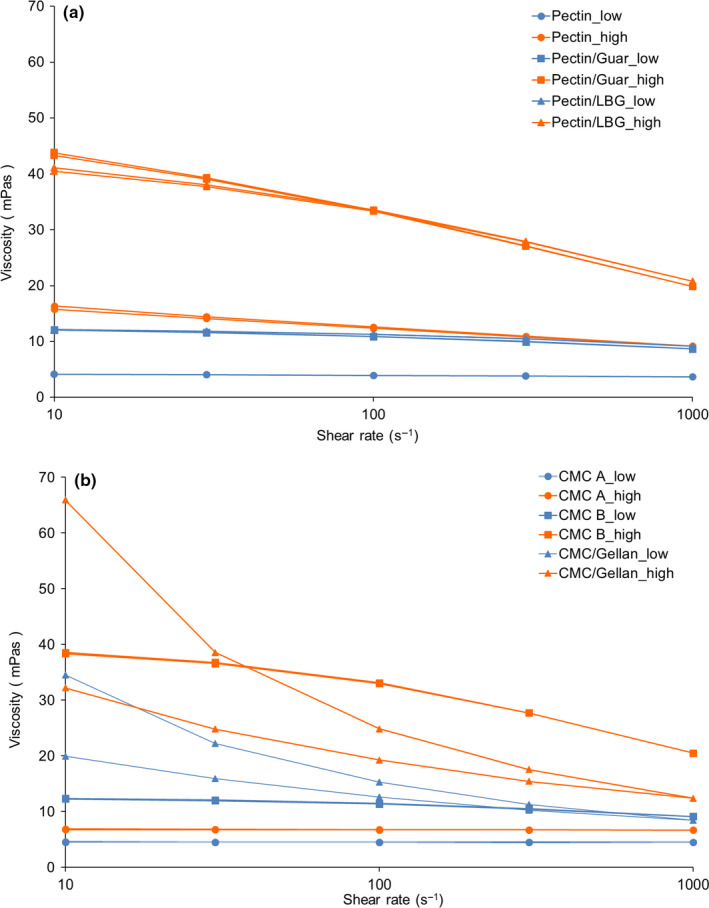
Viscosity of DAPDs added with (a) pectin, pectin/guar gum, and pectin/LBG; (b) CMC A, CMC B, and CMC/gellan gum in low (blue line) and high concentration (orange line). Viscosity was measured with shear rate at 10, 30, 100, 300, 1,000, 300, 100, 30, 10 s^−1^

Figure 2 shows the flow curves of all samples and Table 3 summarizes the rheological parameters by the Power Law model. As seen, the apparent viscosity of samples was increased with increasing concentrations of added stabilizers. Similar results have been obtained in other studies when increasing stabilizer concentrations were used in acidic milk drinks (Arioui et al., 2017; Karimi et al., 2016; Koksoy & Kilic, 2004; Nguyen et al., 2017) and other beverages (Akkarachaneeyakorn & Tinrat, 2015). In the present study, the highest viscosities at a shear rate of 100 s^−1^ in DAPD were achieved by using the mixed stabilizer systems pectin/guar and pectin/LBG at high concentration followed by CMC B and CMC/gellan at high level (the concentrations of the different hydrocolloids are shown in Table 1). Mixed pectin/guar and pectin/LBG resulted in similar viscosities although at low shear the viscosity of pectin/guar was slightly higher. Lowest viscosities were obtained by using only pectin or CMC A. Especially for CMC A, the apparent viscosity increased slightly at a concentration of 0.65% to 0.85%. Research has demonstrated that pectin can form a three‐dimensional network capable of complexing milk components while absorbing maximum water of the medium resulting in an increase in viscosity (Arioui et al., 2017).

**TABLE 3:** Rheological parameters for power law model

Samples	Consistency coefficient (K, mPa s'')	Flow behaviour index (*n*)	*R* ^2^
Pectin_low	4.4	0.97	.992
Pectin_high	22.1	0.88	.997
Pectin/Guar_low	17.3	0.90	.988
Pectin/Guar_high	97.1	0.77	.993
Pectin/LBG_low	17.2	0.91	.981
Pectin/LBG_high	89.3	0.79	.989
CMC A_low	4.6	1.00	.778
CMC A_high	6.9	0.99	.964
CMC B_low	18.3	0.90	.983
CMC B_high	88.1	0.79	.986
CMC/Gellan_low	65.2	0.70	.991
CMC/Gellan_high	138.4	0.64	.989

As seen in Figure 2, the viscosity of samples with high concentrations of stabilizers (except CMC A) decreased with increasing shear rate indicating non‐Newtonian, shear‐thinning behavior as also indicated by the flow behavior indices (*n*) being below 1 (Table 3). This is in agreement with previous research showing that many acidified milk drinks are shear thinning (Janhøj et al., 2008; Karimi et al., 2016) due to the presence of a network structure resulting from protein interaction as well as addition of stabilizer. The lowest flow behavior index and highest consistency coefficient (k) were obtained in the sample with CMC/gellan in high level. In general, samples with a lower concentration of added stabilizer were more close to Newtonian behavior, except in the case of the sample CMC/gellan. According to Laurent and Boulenguer (2003), minimum viscosity and Newtonian behavior will presumably occur when particles ware completed covered by stabilizers.

Another interesting observation was that the viscosity measured in the low shear rate regime was similar during the initial part of the up‐sweep and the final part of the down‐sweep, except for the gellan‐containing samples. These two samples displayed higher initial viscosity (34.50 and 65.93 mPas, respectively, at 10 s^−1^), as compared to the values at the end of the measurement (19.88 and 32.20 mPas). Most likely, this effect was due to the network formed by gellan gum being destroyed during shearing and not being able to reform completely during the timescale of the measurement (Buldo et al., 2016).

### 3.3 Tribological behavior

**FIGURE 3:**
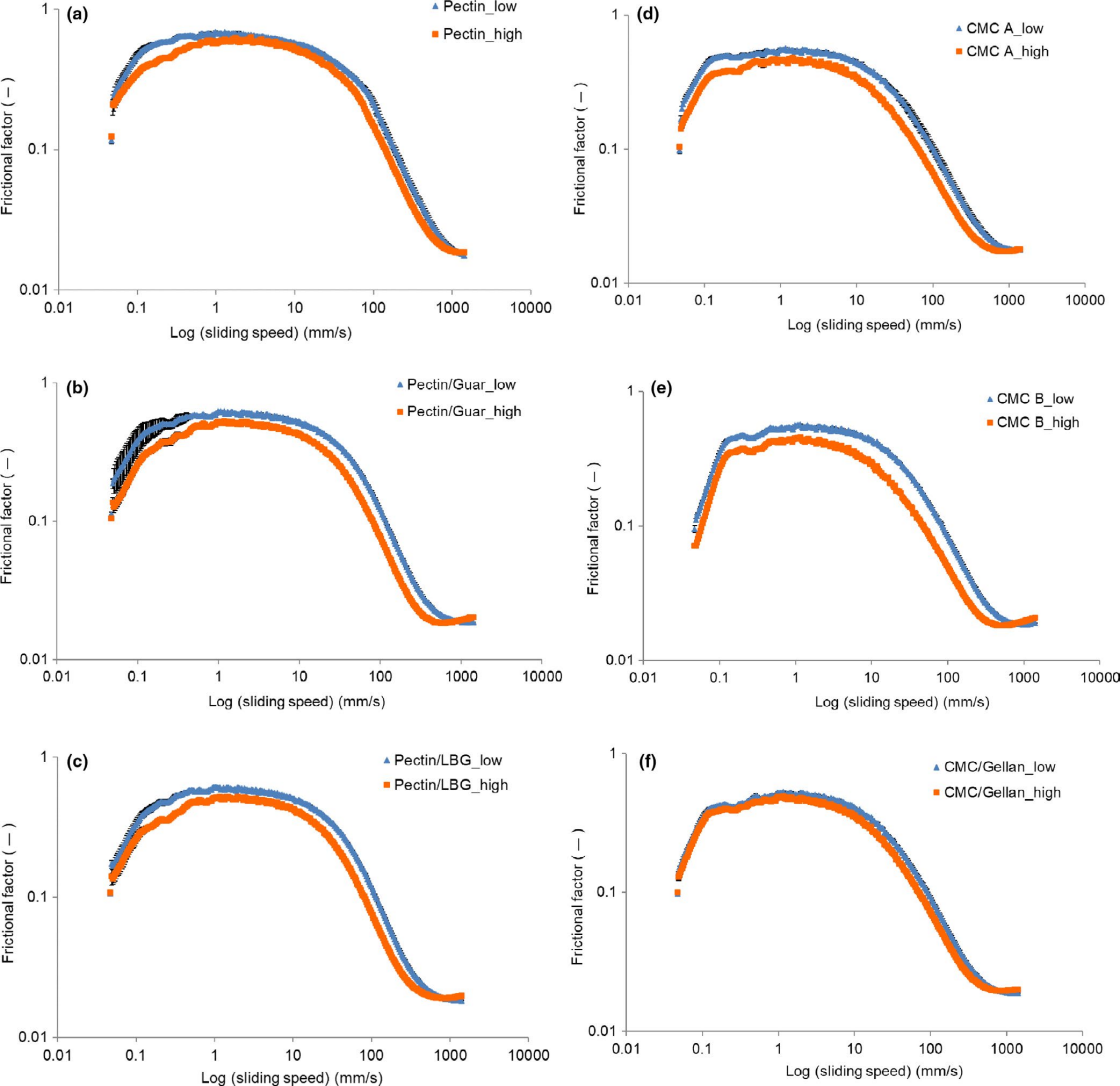
Tribology curves of DAPDs added with pectin, pectin/guar gum, pectin/LBG, CMC A, CMC B, and CMC/gellan gum in low concentration (blue line) and high concentration (orange line)

Lubrication properties (tribological behavior) of all the DAPD samples measured using the tribo‐rheometer at 3N set are presented in Figure 3. As can be seen, the friction curves of all samples show a ‘‘stick and slide’’ pattern (i.e., a traditional Stribeck curve) where the friction coefficient was constant at low sliding speed (0.1–10 mm/s) and decreased with increasing sliding speed (Prakash et al., 2013).

The ‘‘stick and slide’’ pattern is more noticeable for low‐fat products. The reason for this pattern is that at low speeds (boundary regime), the low‐fat product acts as a thin lubricating film and the friction depends on the asperity interaction between the two surfaces while at higher speeds more fluid is drawn into the contact zone to partly separate the two surfaces in the mix regime (Nguyen et al., 2017; Zhu et al., 2019). Interestingly, for all CMC samples, there was a small decline of the friction coefficient prior to reaching the ‘‘stick and slide’’ pattern. This drop could probably relate to initial entrainment of small particles, that is, small aggregates, after an initial liquid regime where particles were not entrained.

Another important observation was that increased concentration of hydrocolloids resulted in lower friction coefficient. Combined with the results in Section 3.3, we can infer that higher viscosity may lead to better lubrication (lower friction coefficient). Similar results were obtained previously by Nguyen et al. (2017) and Zhu et al. (2019), who found that dairy samples with higher viscosity showed lower friction coefficient. The explanation could be that the interaction between dairy protein and hydrocolloids possibly improves the surface adhesion of the resulting solution, allowing formation of a thin film between the two surfaces with ease and hence lowering the friction coefficient.

### 3.4 Sensory evaluation

**FIGURE 4:**
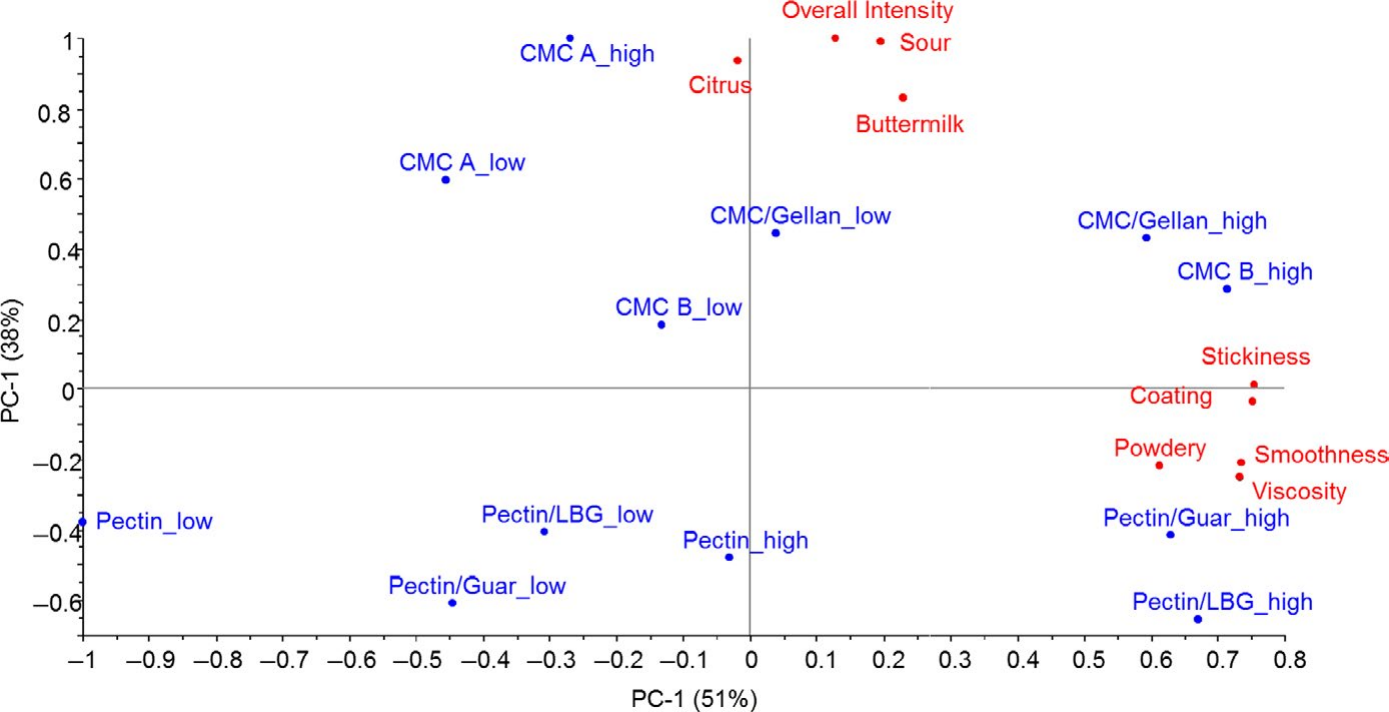
PCA biplot of significant sensory attributes and samples

Table 4 shows the results of the ANOVA analyses on the DA data. All but four attributes (*astringent, sweet, fruity, and metallic*) were found to significantly discriminate between the samples. Figure 4 shows the biplot for the first two principal components, which accounted for 89% of the total variance (51% and 38% for PC1 and PC2, respectively). As seen, increased hydrocolloid resulted in increased sensory viscosity, which is in agreement with the results reported by Nguyen et al. (2017). Highest *viscosities* were obtained in pectin/LBG, pectin/guar, CMC B, and CMC/gellan at high concentration, in accordance with the instrumentally measured viscosity. Strongest sensorial *smoothness*, *stickiness,* and *coating* were also perceived in the aforementioned samples. On the contrary, pectin and CMC_A at low concentration displayed the lowest intensity of *viscosity*, *coating*, *smoothness*, and *stickiness* (Table 4 and Figure 4). The second dimension of the plot was mainly spanned by flavor attributes. All samples with CMC, singly or blended, were more correlated with the sour‐related attributes, for example, *sour*, *citrus,* and *buttermilk* notes. Especially, the sample CMC_A in high level showed the highest *overall intensity* and *sourness*. In contrast, all samples with pectin were less related to the sour‐related flavor attributes and presented lower overall flavor intensity. This could be due to different nature of pectin and CMC: samples with CMC need more acid to reach the same pH as DAPDs with pectin (pH 4.0 in this study) due to a larger buffer effect of CMC.

**TABLE 4:** Mean scores for each sensory attribute in Descriptive Analysis by trained panel

	Pectin_low	Pectin_high	Pectin/	Pectin/	Pectin/	Pectin/	CMC A_low	CMC A_high	CMC B_low	CMC B_high	CMC/	CMC/	Sig^#^
			Guar_low	Guar_high	LBG_low	LBG_high					Gellan_low	Gellan_high	
Viscosity	2.2^e^	7.3^c^	6.3^bc^	11.2^ab^	5.8^cd^	12.1^a^	3.7^e^	4.1^de^	6.2^c^	12.2^a^	6.5^c^	10.1^b^	***
Smoothness	4.0^f^	8.7^bc^	7.6^cde^	10.5^ab^	7.3^cde^	10.8^ab^	5.5^def^	5.8^def^	8.0^cd^	11.3^a^	8.0^cd^	10.4^ab^	***
Stickiness	2.5^f^	5.8^cde^	4.7^def^	10.1^a^	5.3^de^	10.6^a^	4.0^ef^	5.6^cde^	6.9^cd^	10.9^a^	7.6^bc^	9.7^ab^	***
Coating	3.0^d^	7.2^b^	5.2^bcd^	9.8^a^	5.1^bcd^	9.9^a^	3.9^cd^	5.8^bc^	7.2^b^	10.3^a^	7.3^b^	9.7^a^	***
Powdery	1.1^c^	2.4^abc^	1.5^bc^	3.1^a^	2.0^abc^	2.7^ab^	1.9^abc^	1.8^abc^	1.3^bc^	2.1^abc^	1.9^abc^	2.4^abc^	**
Astringent	9.0	8.1	8.0	8.8	9.2	8.0	8.6	9.7	9.0	8.7	8.7	9.1	ns
Sweet	10.7	11.4	11.4	10.7	10.3	10.9	10.7	10.7	10.8	10.7	11.3	11.2	ns
Sour	7.4^e^	7.5^e^	6.9^e^	8.8^bcde^	8.3^cde^	7.9^de^	10.5^ab^	11.3^a^	9.5^abcd^	10.1^abc^	9.9^abc^	10.5^ab^	***
Citrus	8.0^bcd^	8.2^bcd^	7.6^bcd^	8.5^bc^	7.1^cd^	6.3^d^	9.3^ab^	10.8^a^	8.5^bc^	9.3^ab^	9.3^ab^	8.9^abc^	***
Fruity	7.7	9.6	8.9	10.4	8.4	8.4	7.8	7.9	8.6	8.4	9.2	9.1	ns
Buttermilk	4.6^ab^	4.9^ab^	4.2^b^	4.3^b^	4.9^ab^	6.2^ab^	6.4^ab^	6.7^a^	5.8^ab^	6.1^ab^	6.2^ab^	6.4^ab^	**
Metallic	6.9	5.1	5.9	5.5	7.1	4.9	4.8	6.0	4.9	5.3	5.3	5.0	ns
Overall Intensity	8.2^d^	8.4^d^	8.6^cd^	9.2^bcd^	8.9^cd^	8.0^d^	10.8^a^	11.6^a^	10.1^abc^	10.6^ab^	10.8^ab^	11.4^a^	***

^#^ Significance levels: ^***^ *p* < .001, ^**^ *p* < .01, ns = not significant. Within rows, means not sharing superscript letters were significantly different (*p* < .05), following pairwise comparison by Tukey HSD test.

The effects of the stabilizers on flavor have not been studied as much as their effects on texture in acidified milk beverages. Guar gum was reported to cause taste problems and steam treatment was recommended to reduce them (Fox et al., 1993; Seth, Mishra, & Deka, 2018). In the present work, no stabilizer or blend gave a foreign or unacceptable taste. Notably, when blending pectin or CMC with other hydrocolloids, mainly the sensorial viscosity was affected, whereas the flavor perception was only slightly affected. The sensory properties of samples appeared to corroborate that self‐aggregation occurred as a consequence of the addition of mixed hydrocolloids and since the viscosity was much more influenced than the flavor. In several yogurt‐based Iranian drinks incorporated with 0.05 wt % gellan and/or 0.25 wt % pectin, no significant differences in aroma, taste, or mouthfeel were observed (Kiani et al., 2010). Therefore, use of combination of different stabilizers can be investigated to provide an effect at minimum levels not affecting the flavor in acidified milk beverages.

### 3.5 Correlation between sensory evaluation and instrumental measurements

It is not possible to understand complex sensory perceptions with a single instrumental test but inter‐relationships between important sensory textural attributes and mechanical parameters will help in the formulation of right texture foods (van Vliet, 2002). Pearson's correlations, along with significance levels, between sensory and instrumental measurements are displayed in Table 5. Instrumental viscosity at the shear rates of 30 and 100 s^−1^ was used to perform the rheological‐sensory correlation in the current study. As can be seen that, the four sensory texture attributes, that is, *viscosity, smoothness, stickiness,* and *coating* exhibited significantly positive correlations with instrumentally measured viscosity at both 30 and 100 s^−1^ (*r* between 0.92 and 0.97, *p* < .01). These positive correlations have been found for a wide range of food products ranging from Newtonian fluids to thick emulsions (Laiho et al., 2017; Shama & Sherman, 1973; Sonne et al., 2014) and indicate that a more viscous sample will need more energy to spread and swallow. Such knowledge will help develop diets for people with dysphagia. With respect to the tribological‐sensory correlation, it has been reported that the human tongue is estimated to move at speeds up to 200 mm/s (Hiiemae & Palmer, 2003) and thus in the current work the tribology measurements at the range from 0.1 to 100 mm/s were used to associate with sensory variables. Negative correlations were obtained between sensory perceived *viscosity, smoothness, stickiness, coating,* and friction coefficients, with the lowest r values at 0.1 mm/s. Except the correlations between *viscosity, smoothness,* and friction coefficients at 10 mm/s, all the correlations were significant. Moreover, *stickiness* and *coating* showed lower r values compared with *viscosity* and *smoothness*. Negative relationships between perceived *smoothness*, *stickiness* or *coating* and friction have also been found by other researchers (Chojnicka‐Paszun et al., 2014; Laiho et al., 2017).

**TABLE 5:** Correlation coefficients between sensory attributes and instrumental measurements

Sensory attributes	Particle size		Viscosity at shear rate s^−1^		Friction coefficient at speed mm/s			
	D[4,3]	D[3,2]	30	100	0.1	1	10	100
Viscosity	0.17	−0.18	0.96**	0.97**	−0.86**	−0.59*	−0.53	−0.63*
Smoothness	0.19	−0.15	0.92**	0.92**	−0.86**	−0.60*	−0.54	−0.66*
Stickiness	0.36	0.00	0.94**	0.94**	−0.90**	−0.75**	−0.71*	−0.76**
Coating	0.31	−0.07	0.93**	0.92**	−0.89**	−0.73**	−0.66*	−0.70*
Powdery	−0.06	−0.45	0.78	0.77	−0.75**	−0.50	−0.33	−0.47
Sour	0.83**	0.76**	0.04	−0.01	−0.24	−0.75**	−0.81**	−0.65*
Citrus	0.74**	0.64*	−0.20	−0.24	0.00	−0.55	−0.60*	−0.35
Buttermilk	0.56	0.65*	0.08	0.04	−0.24	−0.73**	−0.74**	−0.59*
Overall Intensity	0.85**	0.85**	−0.04	−0.11	−0.13	−0.67*	−0.76**	−0.59*

* *p* < .05.

** *p* < .01.

The flavor attributes, that is, *sour, citrus, buttermilk,* and *overall intensity* were positively correlated to the particle size parameters. This is possibly because the CMC samples had higher titration values and at the same time they generally had higher particle size compared with pectin samples. Negative relationship between flavor attributes and friction coefficient was obtained. Whereas this could be something else than the flavor attributes which affects the correlations. More importantly, it is noteworthy that such existing relationship is only valid within the remits of the specific conditions, for example, the production of DAPDs with these stabilizers. More research is needed to obtain a general mechanical‐sensory relationship.

## 4 CONCLUSIONS

High methoxyl pectin and carboxymethylcellulose (CMC), alone or in combination with guar gum, locust bean gum (LBG), and gellan gum in different concentrations, were used in preparing directly acidified protein drinks. By measuring particle size distribution, rheological behavior, tribological behavior, and sensory properties, it turned out that pectin or CMC was dominant in the stabilizer mixtures. Guar gum and LBG caused a similar viscosity with the same concentration used; gellan increased viscosity by building a network that was easily destroyed by applying shear and only partly recovered when shear was removed. Increasing the concentration of the hydrocolloids resulted in higher viscosity and better lubrication (lower friction coefficient). The sensory *viscosity, smoothness, coating,* and *stickiness* also intensified as the hydrocolloids increased. The type of hydrocolloids had a strong effect on the sensorial texture perception, but the flavor perception was slightly affected. Correlations between sensory attributes and physicochemical parameters were obtained, however, future research will be needed using, for example, incorporation of real or artificial saliva into oral tribology experiments or dynamic sensory testing. This might be beneficial to understand the exact contribution of friction to these complex sensory attributes and allow the development of tribology‐based predictive equations for specific sensory perceptions. Furthermore, use of combination of different stabilizers can be investigated to provide an effect at minimum levels not affecting the flavor in acidified milk beverages.

## CONFLICT OF INTEREST

The authors have declared that no competing interests exist.
